# Dapagliflozin Promotes Neovascularization by Improving Paracrine Function of Skeletal Muscle Cells in Diabetic Hindlimb Ischemia Mice Through PHD2/HIF-1α Axis

**DOI:** 10.3389/fphar.2020.01104

**Published:** 2020-08-10

**Authors:** Dyah Ari Nugrahaningrum, Olivia Marcelina, Caiping Liu, Shourong Wu, Vivi Kasim

**Affiliations:** ^1^ Key Laboratory of Biorheological Science and Technology, Ministry of Education, College of Bioengineering, Chongqing University, Chongqing, China; ^2^ State and Local Joint Engineering Laboratory for Vascular Implants, College of Bioengineering, Chongqing University, Chongqing, China; ^3^ The 111 Project Laboratory of Biomechanics and Tissue Repair, College of Bioengineering, Chongqing University, Chongqing, China

**Keywords:** diabetic HLI, dapagliflozin, skeletal muscle cells, neovascularization, therapeutic angiogenesis

## Abstract

Diabetes mellitus is associated with a high risk of hindlimb ischemia (HLI) progression and an inevitably poor prognosis, including worse limb salvage and mortality. Skeletal muscle cells can secrete angiogenic factors, which could promote neovascularization and blood perfusion recovery. Thus, paracrine function of skeletal muscle cells, which is aberrant in diabetic conditions, is crucial for therapeutic angiogenesis in diabetic HLI. Dapagliflozin is a well-known anti-hyperglycemia and anti-obesity drug; however, its role in therapeutic angiogenesis is unknown. Herein, we found that dapagliflozin could act as an angiogenesis stimulator in diabetic HLI. We showed that dapagliflozin enhances the viability, proliferation, and migration potentials of skeletal muscle cells and promotes the secretion of multiple angiogenic factors from skeletal muscle cells, most plausibly through PHD2/HIF-1α axis. Furthermore, we demonstrated that conditioned medium from dapagliflozin-treated skeletal muscle cells enhances the proliferation and migration potentials of vascular endothelial and smooth muscle cells, which are two fundamental cells of functional mature vessels. Finally, an *in vivo* study demonstrated that intramuscular administration of dapagliflozin effectively enhances the formation of mature blood vessels and, subsequently, blood perfusion recovery in diabetic HLI mice. Hence, our results suggest a novel function of dapagliflozin as a potential therapeutic angiogenesis agent for diabetic HLI.

## Introduction 

In 2014, diabetes mellitus was affecting more than 400 million adults worldwide. Furthermore, more than 640 million adults are predicted to have diabetes in 2040, as the trend keeps increasing (Wiviott et al., 2019b). Diabetes mellitus is generally correlated with microvascular and macrovascular complications, including nephropathy, atherosclerosis, and peripheral arterial disease, which contribute to more than 2 million deaths per annum (Collaboration, 2016). Hindlimb ischemia (HLI) is one of the most prevalent macrovascular complications of diabetes; every 1% increase of hemoglobin A1c contributes to a more than 20% increased risk of developing this disease (Norgren et al., 2007). HLI is indicated by the obstruction of the lower limb arteries, causing ischemic conditions in the lower limbs, which could lead to tissue damage, necrosis, amputation, and even mortality (Annex, 2013). Standard revascularization approaches, such as bypass surgery and vascular stents, which are effective in the majority of non-diabetic HLI patients, are largely inappropriate for diabetic HLI patients due to larger wound surface areas and higher rates of relapse compared to those of non-diabetic patients (Jude et al., 2010; Hoffstad et al., 2015; Ariyanti et al., 2017). Consequently, diabetic patients are posed with a 5-7 times higher major amputation risk (Norgren et al., 2007), raising an emerging need for alternative treatment options for this category of patients. Therapeutic angiogenesis aims to stimulate neovessel formation and blood perfusion in order to alleviate hypoxic damages (Deveza et al., 2012; Annex, 2013). It is regarded as an effective strategy for HLI disease, especially for non-revascularizable patients. However, angiogenesis encompasses a multistep process of stimulating, promoting, and stabilizing new blood vessels, requiring a myriad of angiogenic factors and involving different type of cells (Nussenbaum and Herman, 2010). As such, to date, most angiogenic therapies using angiogenic factors failed to give results as expected (Gupta et al., 2009). Moreover, hyperglycemia disrupts the internal angiogenic potential, creating a larger obstacle in applying therapeutic angiogenesis for diabetic HLI patients (Howangyin and Silvestre, 2014).

Skeletal muscle cells have the ability to release paracrine signals which can improve cell-cell communication among different types of cells, including those involved in the angiogenesis process (Tateno et al., 2006; Karsenty and Olson, 2016; Giudice and Taylor, 2017; Zhang et al., 2017). Due to these characteristics, previous studies have shown that skeletal muscle cells could serve as a potential target for therapeutic angiogenesis (Tateno et al., 2006; Perez-Ilzarbe et al., 2008). Indeed, previous studies have shown that targeting skeletal muscle cells could effectively induce neovascularization in HLI mice (Tateno et al., 2006; Zhang et al., 2017). However, a hyperglycemic condition in diabetic patients significantly impairs the viability and angiogenesis functioning of skeletal muscle cells, as well as those of other fundamental blood vessel-forming cells, including vascular endothelial and smooth muscle cells (Ariyanti et al., 2017; Meza et al., 2019). Hence, restoring these skeletal muscle cells’ potentials are pivotal for utilizing therapeutic angiogenesis in diabetic HLI.

Dapagliflozin is an anti-hyperglycemic oral drug that has been approved by the FDA. It acts by selectively inhibiting sodium-glucose cotransporter 2 (SGLT2) (Dandona et al., 2017; Van Raalte and Cherney, 2018). Previously, Chang *et al.* showed that dapagliflozin protects mouse kidney from ischemic reperfusion injury by inducing hypoxia-inducible factor-1α (HIF-1α) (Chang et al., 2016), a key regulator of multiple angiogenic factors in response to hypoxia. Under normoxia, prolyl hydroxylase domain 2 (PHD2) adds hydroxyl groups to proline-402 and -564 residues of HIF-1α by utilizing oxygen as its substrate. This subsequently leads to HIF-1α protein degradation through ubiquitin/proteasomal degradation pathway (Berra et al., 2003; Appelhoff et al., 2004; Wu et al., 2008). Previous studies have also confirmed that stabilization of HIF-1α could lead to effective angiogenesis in HLI (Patel et al., 2005; Wu et al., 2008; Semenza, 2009). However, whether dapagliflozin could effectively induce neovascularization in diabetic HLI is still unknown.

Herein, we found that dapagliflozin could promote skeletal muscle cells’ viability and migration potential under hyperglycemia, most plausibly by suppressing PHD2, thereby promoting HIF-1α protein stability. Furthermore, accumulation of HIF-1α subsequently increases the skeletal muscle cells’ paracrine potential, leading to an increase of angiogenic factors secretion. This further enhances cell-cell communication between skeletal muscle cells and cells forming the blood vessels, *i.e.*, vascular endothelial and smooth muscle cells, and induces their proliferation and migration potentials. Finally, we show that intramuscular administration of dapagliflozin significantly enhances the formation of mature vessels, as well as blood perfusion recovery in diabetic HLI mice, without affecting the blood glucose concentration. Hence, our study reveals an unknown role of intramuscularly-injected dapagliflozin in direct induction of neovascularization in diabetic HLI, suggesting the potential application of dapagliflozin as a therapeutic angiogenesis strategy for diabetic HLI.

## Materials and Methods

### Cell Lines

C2C12, HUVECs, and MOVAS cell lines were purchased from the American Type Culture Collection. Cells were cultured in Dulbecco’s modified Eagle medium (DMEM; Gibco, Life Technologies, Grand Island, NY) added with 10% fetal bovine serum (FBS; Biological Industries, Beit Haemek, Israel). Regular detection for mycoplasma was performed by Mycoplasma Detection Kit-QuickTest (Biotool, Houston, TX). A hyperglycemic condition was obtained by culturing the cells in DMEM with a final glucose concentration of 25 mM.

### Dapagliflozin Treatment

Dapagliflozin was obtained from Selleckchem Co., Ltd (Shanghai, China; molecular weight: 408.87; purity 99.31%). For dapagliflozin treatment, cells were treated for 24 h with various doses (final concentrations: 2 μM, 10 μM, and 50 μM) of dapagliflozin dissolved in 10% dimethylsulfoxide (DMSO). As the control, an equal volume of DMSO was used. All conditions were performed under the hyperglycemic condition, as described previously. After dapagliflozin treatment, cells were serum-starved by culturing them in FBS-free DMEM under hypoxic condition (Anaeropouch Box, 0.1% O2, Mitsubishi Gas Chemical, Tokyo, Japan).

For *HIF-1α* silencing, C2C12 cells were seeded prior to transfection with control vector (shCon) or shRNA-expressing vector targeting *HIF-1α* (shHIF-1α) using Lipofectamine 2000 (Invitrogen Life Technologies, Grand Island, NY). Twenty-four hours after transfection, antibiotic selection for removing untransfected cells was carried by using puromycin of 2.5 mg/ml for 48 h.

For HIF-1α inhibition, 2-methoxyestradiol was used as the inhibitor drug. Cells were treated with 2-methoxyestradiol (2-ME2; Shanghai Macklin Biochemical, Shanghai, China; final concentration: 10 μM) for 12 h, followed by dapagliflozin treatment as described above. Cells were then exposed to serum starvation and hypoxia.

### Plasmids and Constructs

Murine HIF-1α (NM_001313919) shRNA expression vector (shHIF-1α) was constructed as previously described (Miyagishi and Taira, 2003). The RNAi target site is as follows: GTGAAAGGATTCATATCTA. As a control vector (shCon), a vector expressing a stretch of 7 thymines terminator sequences exactly downstream to the U6 promoter was used.

### Animal Experiment

Male C57BL/6 mice aged 8 weeks (body weight 20-30 g) were obtained from the Third Military Medical University (Chongqing, China). Experiments were done in the Third Military Medical University (Chongqing, China) with consent from the Laboratory Animal Welfare and Ethics Committee of the Third Military Medical University. Anesthesia was done through the administration of ketamine (80 mg/kg) and xylazine (50 mg/kg) intraperitoneally.

For diabetes induction, mice were given a high fat diet for 3 weeks (20% kcal protein, 20% kcal carbohydrate, and 60% kcal fat). Administration of 40 mg/kg body weight streptozotocin (Sigma Aldrich, St. Louis, MO) in sodium citrate buffer intraperitoneally was then performed for the following five days. Mice were fasted overnight prior to each streptozotocin injection and blood glucose level measurement. Blood glucose level was evaluated using Accu-Check Integra (Roche Diagnostics, Shanghai, China). Diabetic mice with blood glucose level ≥ 16 mmol/L were used for HLI establishment.

Before HLI induction, mice were anesthetized as previously described (Zhang et al., 2017). Surgery was performed by ligating and excising the femoral artery of the left limb, while the right limb was left without surgery and used as a control. Mice were then grouped randomly. In the dapagliflozin-treated group (n = 7), dapagliflozin (10 mg/kg body weight) was administered into the gastrocnemius muscle of the left limb, while in the control group (n = 7), PBS was injected correspondingly. Treatments were done every three days for 21 days, starting from twenty-four hours post-surgery.

Damage caused by ischemia was evaluated with visual examination and scored as described previously (0 = no difference with control, 1 = mild change in color, 2 = moderate change in color, 3 = severe change in color, necrosis, loss of subcutaneous tissue, and 4 = lower-extremity amputation) at indicated time points (Stabile et al., 2003; Zhang et al., 2017). Blood perfusion of the lower limb was visualized and analyzed by a Laser Doppler Imager (Moor Instruments Ltd, Axminster, Devon, England) at the indicated times. Prior to visualization, mice were anesthetized and the hair of the lower hindlimb area was depilated. Blood perfusion ratio was acquired by calculating the ratio between ischemic hindlimb (left) to corresponding control (right hindlimb) as described previously (Tanii et al., 2006; Wu et al., 2015; Zhang et al., 2017).

### Preparation of Conditioned Medium

C2C12 cells were treated with dapagliflozin for 24 h under hyperglycemia (final concentration of glucose: 25 mM). Following the treatment, cells were washed, serum-starved, and exposed to hypoxia for 24 h. Conditioned medium was obtained by collecting and filtering the medium with 0.22 μm filter. Conditioned medium from dapagliflozin-treated cells was indicated as CM-Dapa, while conditioned medium from cells treated with same volume of DMSO was indicated as CM-Con.

### Enzyme-Linked Immunosorbent Assay (ELISA)

Secreted amounts of VEGF-A and FGF2 in conditioned medium were determined using Mouse VEGF ELISA kit (Neobioscience, Shenzhen, China) and Mouse bFGF/FGF2 ELISA kit (Elabscience, Wuhan, China), respectively. ELISA was performed according to the manufacturer’s guidelines.

### Cell Viability and Crystal Violet Staining

C2C12 cells were treated with the indicated doses of dapagliflozin for 24 h in the hyperglycemic condition (final concentration of glucose: 25 mM). Following the treatment, cells were reseeded in a 96-well plate and exposed to hypoxia at indicated times. Cell proliferation assay was performed by using an MTS reagent kit (Promega, Madison, WI) for 2 h. Viable cell numbers were measured with a spectrophotometric microplate reader (BioTek Instruments, Winooski, VT) at a wavelength of 490 nm. For crystal violet staining, C2C12 cells were seeded in a 24-well plate and treated with indicated doses of dapagliflozin for 24 h. Following 24 h exposure to hypoxia, cells were fixed by 5 min incubation with 4% paraformaldehyde (PFA) and stained for 30 min with 0.05% crystal violet (Beyotime, Shanghai, China).

### EdU Incorporation Assay

Cells were treated with dapagliflozin under hyperglycemia (final concentration of glucose: 25 mM) for 24 h and then exposed to hypoxia for 12 h. EdU incorporation assay was performed using BeyoClick™ EdU-488 Cell Proliferation Assay Kit (Beyotime, Shanghai, China) according to the manufacturer’s instruction. Images were obtained using Olympus IX73 (Olympus, Tokyo, Japan). Quantification was done by using Image J software and results were shown as the ratio of cells between those of EdU-positive to Hoechst-positive. For experiments using the conditioned medium, HUVECs and MOVAS cells were cultured with CM-Con or CM-Dapa prior to exposure to hypoxia and EdU incorporation assay.

### Western Blotting

Cells were treated with indicated doses of dapagliflozin under hyperglycemia (final concentration of glucose: 25 mM) for 24 hours. Following the treatment, cells were exposed to hypoxia for 12 hours prior to protein extraction and western blotting experiment. For the animal study, frozen gastrocnemius muscle of the ischemic limb was obtained and used for protein tissue extraction for western blot analysis. The detailed method for western blotting was described in a previous study (Wu et al., 2018). Antibodies used in western blotting are listed in **Table S1**. β-actin was used as the loading control. Protein quantification was analyzed with Quantity One software (Thermo Scientific, Waltham, MA). Data were represented as the relative expression of protein in the treatment group compared to the control, which was assumed as 1.

### RNA Extraction and Quantitative Reverse Transcription PCR (qRT-PCR)

C2C12 cells were treated with 10 μM dapagliflozin or an equal volume of DMSO under hyperglycemia (final concentration of glucose: 25 mM) for 24 h. After the treatment, cells were serum-starved and exposed to hypoxia for 6 h, then total RNA was extracted using Trizol (Invitrogen Life Technologies) according to manufacturer’s instruction. Total RNA (1 μg) was reverse-transcribed into cDNA using the PrimeScript RT Reagent Kit with gDNA Eraser (Takara Bio, Dalian, China). qRT-PCR analysis of cDNA samples was performed using SYBR Premix Ex Taq (Takara Bio) to analyze the mRNA expression levels. The sequences of MyoD1 (NM_010866) primer pair are as follows: forward primer: AGCACTACAGTGGCGACTCA; reverse primer: GGCCGCTGTAATCCATCAT. The sequences of MyoG (NM_031189) are as follows: forward primer: CCTTGCTCAGCTCCCTCA; reverse primer: TGGGAGTTGCATTCACTGG. β-Actin (NM_007393) was used for normalization of sample amplification and its primer pair sequences are as follows: forward primer: AGATGTGGATCAGCAAGCAG; reverse primer: GCGCAAGTTAGGTTTTGTCA. Results were shown as a relative expression of mRNA level to the control, which was assumed as 1.

### Immunofluorescence Staining

For immunofluorescence staining, frozen gastrocnemius muscle from the ischemic limb of diabetic mice was sliced at 10 μm thickness and stained for PECAM-1 and α-SMA antibodies, as described previously (Zhang et al., 2017). Briefly, the tissue sections were incubated with PECAM-1 antibody for 1 hour. Afterwards, the tissue was incubated with monoclonal antibody against murine α-SMA conjugated with Cy3 and Alexa Fluor 488 Goat Anti-Rat IgG. Antibodies used in immunofluorescence staining are listed in **Table S1**. Images were obtained with Microsystems-TPS SP8 (Leica, Heidelberg, Germany).

### Scratch Assay

Cells were seeded in a 6-well plate (2x10^5^ cells/each well) and treated with dapagliflozin or the same volume of DMSO under hyperglycemia (final concentration of glucose: 25 mM), followed by exposure to hypoxic condition at indicated times. After changing the culture medium with culture medium without dapagliflozin, cyclohexamine (final concentration: 10 mg/ml, purity ≥ 95%, Cayman Chemicals, Ann Arbor, MI) was added to the culture medium to stop cell proliferation. Cells were scratched in a straight line and placed in hypoxic condition.

### Transwell Migration Assay

Cells were treated with 10 μM dapagliflozin or the same volume of DMSO under a hyperglycemic condition (final concentration of glucose: 25 mM) for 24 h. Following the treatment, cells were re-seeded (8x10^3^ per well) into the upper compartment of the transwell chamber (Corning, NY, USA) and exposed to hypoxia for 24 h. Afterwards, cells migrated into the lower compartment and were stained using crystal violet (Beyotime). Visualization was employed using Olympus IX71 (Olympus). For experiments using the conditioned medium, HUVECs and MOVAS cells were seeded into the upper compartment, while the conditioned medium was placed in the lower compartment of the transwell chamber prior to exposure to hypoxia.

### Phalloidin Staining

Cells seeded in a glass bottom dish (9x10^3^ per well) were treated with 10 μM dapagliflozin under hyperglycemia (final concentration of glucose: 25 mM) for 24 h. Subsequent to the treatment, cells were incubated under hypoxia for 12 h, as described above. Prior to the staining, cell fixation and permeabilization were employed by incubating the cells with 4% PFA and 0.1% Triton X-100, respectively. Cells were then incubated with 1% bovine serum albumin, followed by Phalloidin at room temperature for 60 min each. Cell imaging was obtained with Microsystems-TPS SP8 (Leica). Fractal dimension analysis was done using Image J software. For the conditioned medium experiment, HUVECs and MOVAS cells were cultured in the conditioned medium for 24 h, in advance to hypoxia induction for 12 h.

### Statistical Analysis

Statistical analysis for the blood perfusion ratio between time points *in vivo* was carried out by using repeated-measures ANOVA, while difference between treatment groups was evaluated using one-way ANOVA. *In vivo* limb morphological assessment analysis was performed by a nonparametric Mann-Whitney test. Other statistical analysis was performed using Student’s *t* test. *P < 0.05 was considered as significantly different, while high significance was defined as **P < 0.01.

## Results

### Dapagliflozin Improves Skeletal Muscle Cells Viability and Proliferation

**Figure 1:**
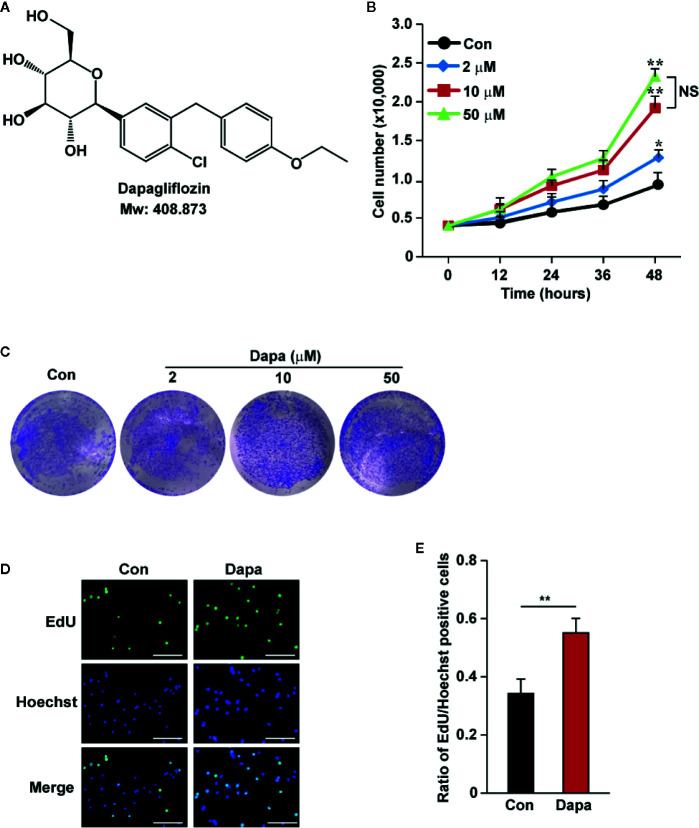
Dapagliflozin induces skeletal muscle cells’ proliferation and viability under hyperglycemia. **(A)** Chemical structure of dapagliflozin. **(B)** Total number of C2C12 cells after treatment with different doses of dapagliflozin at indicated time points (n = 3). **(C)** The quantity of C2C12 cells after treatment with dapagliflozin at indicated doses, as examined using crystal violet staining (n = 3). **(D, E)** The effect of dapagliflozin treatment (final concentration: 10 μM) on C2C12 cells proliferation potential as evaluated by EdU incorporation assay: **(D)** representative images (scale bars: 100 μm) and **(E)** ratio of EdU-positive cells to Hoechst-positive cells (n = 6) were shown. All experiments were done under hyperglycemic and hypoxic condition. Cells treated with DMSO were used as controls. Data were presented as mean ± SD. NS: not significant, *P < 0.05, **P < 0.01; Con: DMSO-treated C2C12 cells, Dapa: dapagliflozin-treated C2C12 cells.

Hyperglycemia could induce pathological damage, such as decreased cell viability and proliferation in affected cells and tissues, leading to defective angiogenesis induction and poor prognosis (Howangyin and Silvestre, 2014; Moriya and Ferrara, 2015; Ariyanti et al., 2019). Dapagliflozin (**Figure 1A**) is the first SGLT2 inhibitor used for anti-hyperglycemic therapy. It exerts protective roles in diabetic renal function by suppressing apoptosis; however, whether it could be applied for therapeutic angiogenesis in diabetic HLI remains unknown. To elucidate this possibility, first we investigated whether dapagliflozin could increase skeletal muscle cells’ viability and proliferation potentials under hyperglycemia. As shown in **Figures 1B, C**, we found that dapagliflozin treatment could significantly increase the total cell number of C2C12 in a dose-dependent manner. It is noteworthy that treatment with 10 μM dapagliflozin was sufficient to yield a significant effect, while there was no significant difference between treatments with 10 μM and 50 μM dapagliflozin at 48 h. Hence, we used 10 μM dapagliflozin for further analysis. Moreover, dapagliflozin also increased the ratio of EdU-positive cells, indicating its potential to enhance skeletal muscle cell proliferation (**Figures 1D, E**). Furthermore, in response to ischemic injury, skeletal muscle cells underwent a regeneration process by proliferating and differentiating to form muscle fibers (Charge and Rudnicki, 2004). However, hyperglycemia is known to disrupt the differentiation potential of skeletal muscle cells (Brannon et al., 1989; Kozakowska et al., 2015). Our results showed that dapagliflozin could significantly induce the mRNA expression levels of myogenic differentiation 1 (MyoD1) and myogenin (MyoG) (**Figure S1**), which are two myogenic regulatory factors involved in the early and later stages of skeletal muscle differentiation, respectively (Yun et al., 2005). These results indicated that dapagliflozin could promote skeletal muscle viability and proliferation, as well as differentiation potentials, under hyperglycemia.

### Dapagliflozin Activates Angiogenic Factors Expression in Skeletal Muscle Cells

**Figure 2:**
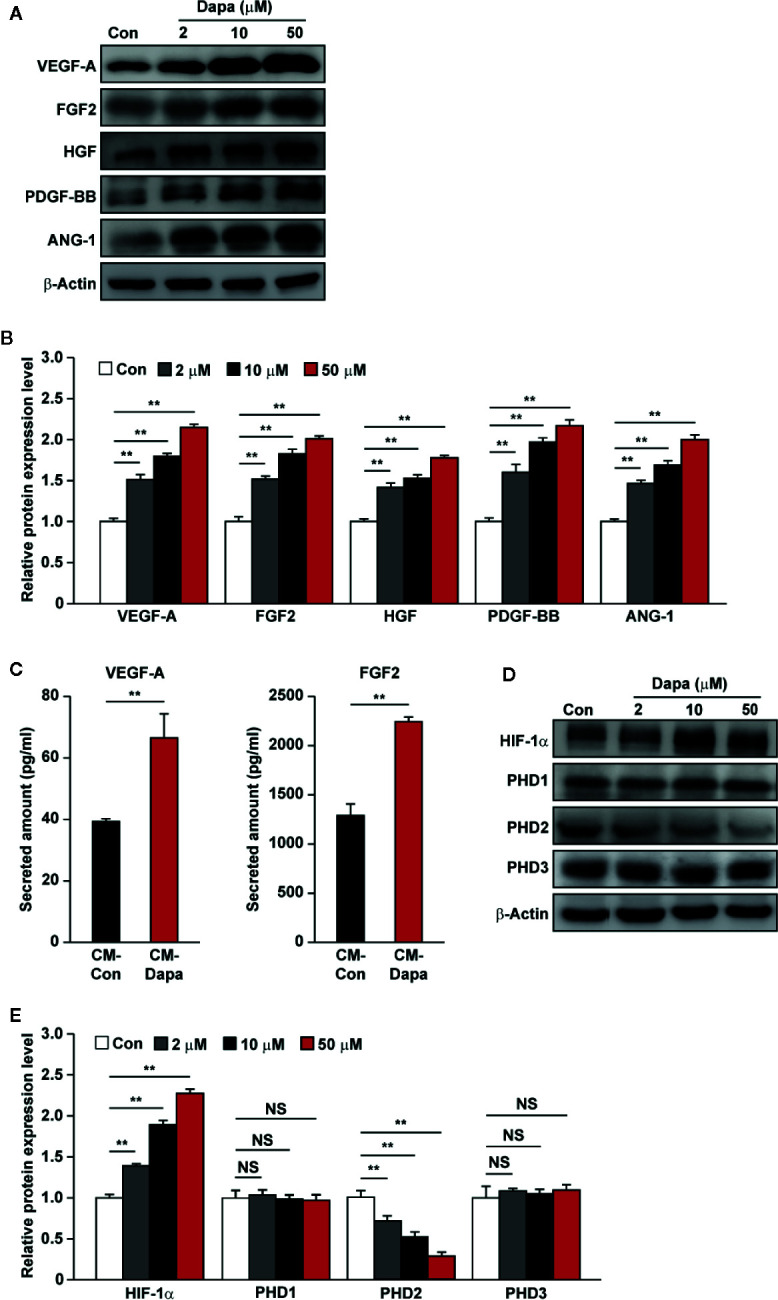
Dapagliflozin promotes the protein expression levels of angiogenic factors in skeletal muscle cells under hyperglycemia. **(A, B)** The protein levels of angiogenic factors in C2C12 cells treated with indicated doses of dapagliflozin. Protein levels were examined using western blotting: **(A)** representative images and **(B)** quantification results were shown. **(C)** Secretion levels of VEGF-A and FGF2 in C2C12 cells treated with dapagliflozin (final concentration: 10 μM), as determined by ELISA. **(D, E)** The protein levels of angiogenic factor regulators in C2C12 cells treated with indicated doses of dapagliflozin. Protein levels were examined using western blotting: **(D)** representative images and **(E)** quantification results were shown. All experiments were done under hyperglycemic and hypoxic conditions. Cells treated with DMSO were used as controls. β-actin was used as a loading control for western blotting. Data was quantified as relative to controls and presented as mean ± SD (n = 3). NS, not significant, **P < 0.01; Con: DMSO-treated C2C12 cells, Dapa: dapagliflozin-treated C2C12 cells.

Angiogenesis involves a concert of signaling cascades exerted by numerous angiogenic factors. Previously, our studies found that, while skeletal muscle cells are capable of secreting these angiogenic factors, hyperglycemia disturbs this function (Ariyanti et al., 2017; Zhang et al., 2017). Thus, we analyzed whether dapagliflozin could enhance the angiogenic function of skeletal muscle cells. Protein levels of VEGF-A, FGF2, HGF, PDGF-BB, and ANG-1 in dapagliflozin-treated C2C12 cells showed significant increases (**Figures 2A, B**). Concomitantly, the secretion levels of VEGF-A and FGF2 from dapagliflozin-treated cells also increased robustly (**Figure 2C**).

To investigate the molecular mechanism of dapagliflozin regulation on the expression levels of angiogenic factors in the skeletal muscle cells, we investigated the effect of dapagliflozin treatment on the expression level of HIF-1α, a key regulator of the angiogenic process which could activate the transcription of numerous factors, including VEGF-A and PDGF-BB (Kelly et al., 2003). We found that dapagliflozin treatment increased the accumulation of HIF-1α protein (**Figures 2D, E**). Furthermore, as shown in **Figure 2D**, dapagliflozin treatment suppressed the level of PHD2, a negative regulator that could induce HIF-1α degradation (Berra et al., 2003). PHD2 inhibition could also induce the expression of angiogenic factors, such as HGF, FGF2, ANG1, and HIF-1α-independently (Onimaru et al., 2002; Wu et al., 2008; Chan et al., 2009; Wu et al., 2015). Intriguingly, dapagliflozin did not affect the levels of other PHD family members, that is, PHD1 and PHD3. Collectively, these results suggested that dapagliflozin treatment could promote angiogenic factors’ expression and secretion through suppressing PHD2 expression, which in turn increases HIF-1α protein accumulation in skeletal muscle cells.

### Dapagliflozin Promotes Skeletal Muscle Cells Migration Potential

**Figure 3:**
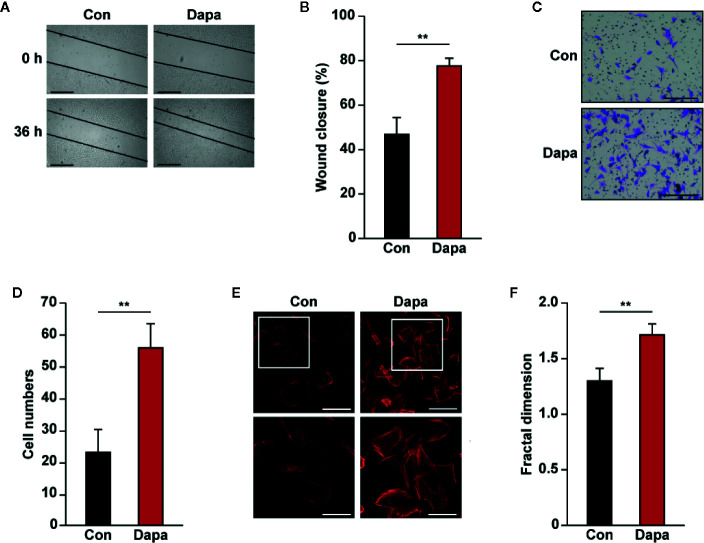
Dapagliflozin enhances skeletal muscle cells’ migration potential under hyperglycemia. **(A, B)** Migration potential of C2C12 cells after dapagliflozin treatment (final concentration: 10 μM), as analyzed by scratch assay: **(A)** representative images scale bars: 200 μm) and **(B)** quantification of wound closure rate (n = 6) were shown. **(C, D)** Migration potential of C2C12 cells after dapagliflozin treatment (final concentration: 10 μM), as investigated using transwell migration assay: **(C)** representative images (scale bars: 100 μm) and **(D)** quantification results (n = 6) were shown. **(E, F)** Polymerization of F-actin in C2C12 cells treated with dapagliflozin (final concentration: 10 μM), as analyzed using phalloidin staining: **(E)** representative images (scale bars: 100 μm for upper panels and 50 μm for lower panels) and **(F)** quantification of fractal dimension (n = 6) were shown. All experiments were done under hyperglycemic and hypoxic conditions. Cells treated with DMSO were used as controls. Quantification data were presented as mean ± SD. **P < 0.01; Con: DMSO-treated C2C12 cells, Dapa: dapagliflozin-treated C2C12 cells.

Skeletal muscle cell migration has an important role for neovascularization, as it enables secreted angiogenic factors to be dispersed into larger ischemic areas (Zhang et al., 2017). We further analyzed whether dapagliflozin could enhance the migration potential of skeletal muscle cells. The scratch assay demonstrated that dapagliflozin improves cell migration (**Figures 3A, B**). In line with this, dapagliflozin enhanced the number of C2C12 cells migrated into the lower compartment of the transwell chamber, further confirming the stimulatory effect of dapagliflozin on skeletal muscle cells’ migration potential (**Figures 3C, D**). Polymerization of F-actin from G-actin is necessary for cell movement; hence, the increase of F-actin is one of the indicators of enhanced cell migration potential. As shown in **Figures 3E, F**, phalloidin staining results showed that dapagliflozin promoted F-actin polymerization, indicating that dapagliflozin enhanced cellular migration potential most plausibly by inducing F-actin polymerization.

### HIF-1α Mediates the Effect of Dapagliflozin on Skeletal Muscle Cells

**Figure 4:**
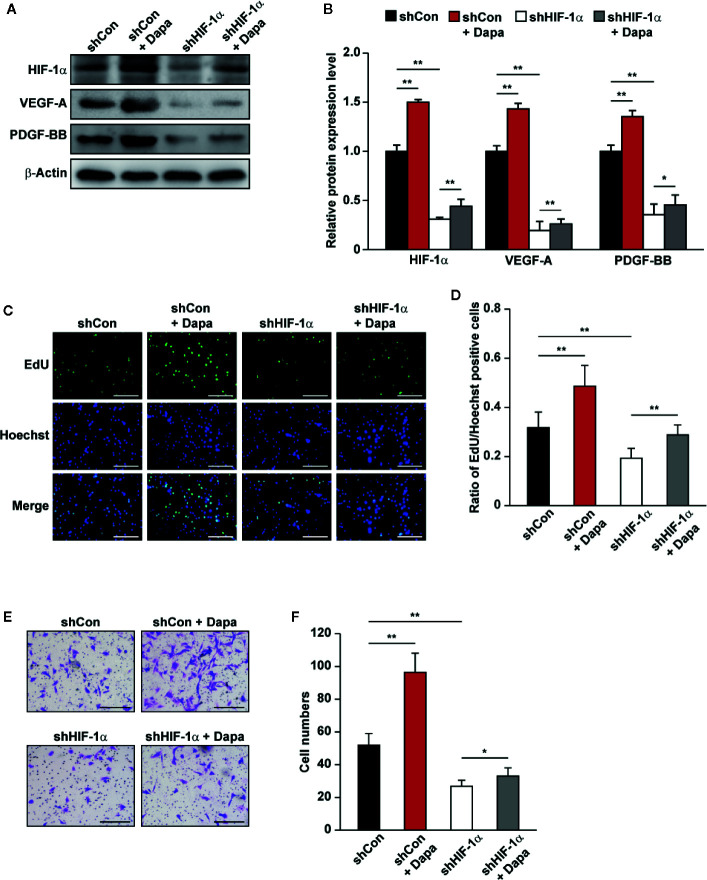
Dapagliflozin promotes skeletal muscle cells’ angiogenesis potentials through HIF-1α. **(A, B)** The protein levels of angiogenic factors in *HIF-1α*-silenced C2C12 cells treated with dapagliflozin (final concentration: 10 μM). Protein levels were examined using western blotting: **(A)** representative images and **(B)** quantification results were shown (n = 3). **(C, D)** The effect of dapagliflozin treatment (final concentration: 10 μM) on *HIF-1α*-silenced C2C12 cells’ proliferation potential as evaluated by EdU incorporation assay: **(C)** representative images (scale bars: 100 μm) and **(D)** ratio of EdU-positive cells to Hoechst-positive cells (n = 6) were shown. **(E, F)** The effect of dapagliflozin (final concentration: 10 μM) on the migration potential of *HIF-1α*-silenced C2C12 cells, as investigated using transwell migration assay: **(E)** representative images (scale bars: 100 μm) and **(F)** quantification results (n = 6) were shown. All experiments were done under hyperglycemic and hypoxic conditions. Cells treated with DMSO were used as controls. β-actin was used as a loading control for western blotting. Data was quantified as relative to controls and presented as mean ± SD. *P < 0.05, **P < 0.01; shCon: shCon-transfected C2C12 cells treated with DMSO, shCon+Dapa: shCon-transfected C2C12 cells treated with dapagliflozin, shHIF-1α: *HIF-1α*-silenced C2C12 cells treated with DMSO, shHIF-1α+Dapa: *HIF-1α*-silenced C2C12 cells treated with dapagliflozin.

**Figure 5:**
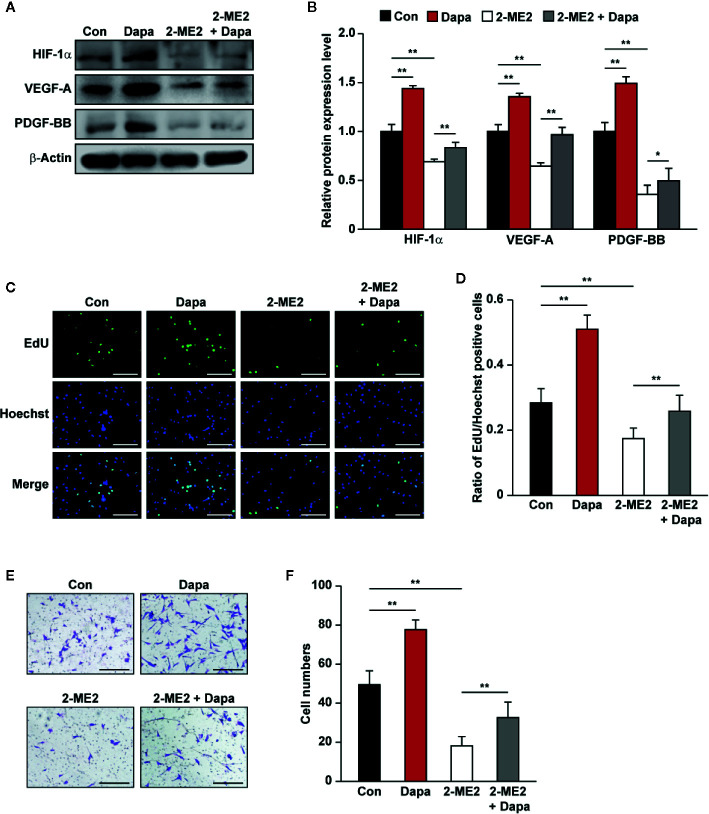
Dapagliflozin enhances skeletal muscle cells’ angiogenesis potentials through HIF-1α. **(A, B)** The protein levels of angiogenic factors in C2C12 cells treated with 2-ME2 and dapagliflozin (final concentration: 10 μM). Protein levels were examined using western blotting: **(A)** representative images and **(B)** quantification results (n = 3) were shown. **(C, D)** The effect of dapagliflozin treatment (final concentration: 10 μM) on 2-ME2-treated C2C12 cells’ proliferation potential as evaluated by EdU incorporation assay: **(C)** representative images (scale bars: 100 μm) and **(D)** ratio of EdU-positive cells to Hoechst-positive cells (n = 6) were shown. **(E, F)** Migration potential of C2C12 cells after 2-ME2 and dapagliflozin treatment (final concentration: 10 μM), as investigated using transwell migration assay: **(E)** representative images (scale bars: 100 μm) and **(F)** quantification results (n = 6) were shown. All experiments were done under hyperglycemic and hypoxic conditions. Cells treated with DMSO were used as controls. β-actin was used as a loading control for western blotting. Data was quantified as relative to controls and presented as mean ± SD. *P < 0.05, **P < 0.01; Con: DMSO, Dapa: dapagliflozin, 2-ME2: 2-methoxyestradiol.

PHD2 suppression in skeletal muscle cells has been shown to enhance HIF-1α accumulation and angiogenic factors’ expression (Wu et al., 2015; Settelmeier et al., 2020). As the downstream factors of HIF-1α, VEGF-A and PDGF-BB could exert an autocrine signaling to stimulate skeletal muscle cells’ proliferation and migration potentials (Webb and Lee, 1997; Germani et al., 2003). The fact that dapagliflozin robustly increased the accumulation of HIF-1α protein, while suppressing PHD2 expression, suggested that dapagliflozin might exert its effect on skeletal muscle cells through PHD2/HIF-1α axis. To further confirm the role of HIF-1α in mediating the dapagliflozin effect, we examined the effect of dapagliflozin on *HIF-1α*-silenced C2C12 cells. Indeed, *HIF-1α* silencing abolished the stimulatory effect of dapagliflozin on the levels of VEGF-A and PDGF-BB in the skeletal muscle cells (**Figures 4A, B**). In line with this, *HIF-1α* silencing could diminish the dapagliflozin-induced proliferation (**Figures 4C, D**) and migration potentials of skeletal muscle cells (**Figures 4E, F**). Similar effects were observed when C2C12 cells were treated with 2-ME2, a HIF-1α inhibitor, as dapagliflozin-induced angiogenic factors expression in C2C12 cells were suppressed when HIF-1α was inhibited (**Figures 5A, B**). As shown in **Figures 5C–F**, dapagliflozin stimulatory effects on skeletal muscle cells proliferation, as well as on cell migration potential, significantly decreased upon treatment with 2-ME2. Overall, these results indicated the importance of HIF-1α in mediating the pro-angiogenic effect of dapagliflozin on skeletal muscle cells.

### Dapagliflozin Increases the Proliferation and Migration Potentials of Vascular Endothelial and Smooth Muscle Cells *via* Skeletal Muscle Cells Secretome

**Figure 6:**
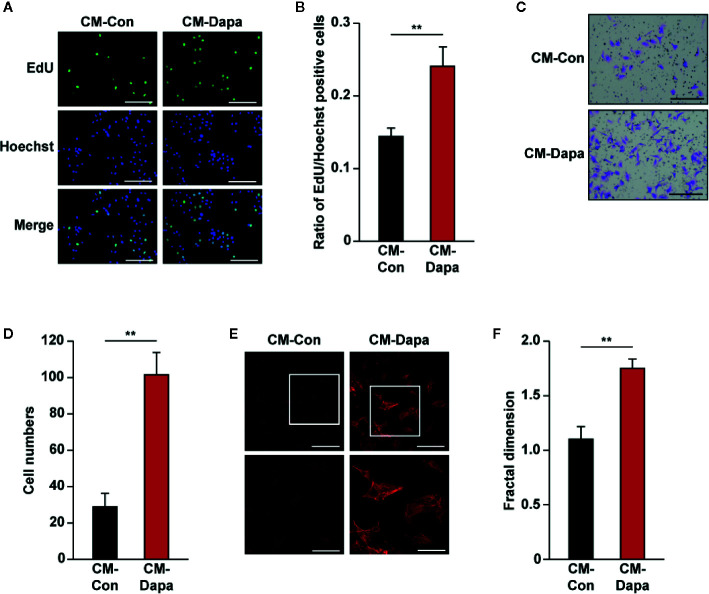
Dapagliflozin enhances vascular endothelial cells’ migration and proliferation potentials under hyperglycemia through secretome of skeletal muscle cells. **(A, B)** The effect of CM-Dapa on HUVECs proliferation potential, as examined using EdU incorporation assay: **(A)** representative images (scale bars: 100 μm) and **(B)** ratio of EdU-positive cells to Hoechst-positive cells (n = 6) were shown. **(C, D)** The effect of CM-Dapa on HUVECs migration potential, as analyzed using transwell migration assay: **(C)** representative images (scale bars: 100 μm) and **(D)** quantification results (n = 6). **(E, F)** Polymerization of F-actin in HUVECs cultured with CM-Dapa, as analyzed using phalloidin staining: **(E)** representative images (scale bars: 100 μm for upper panels and 50 μm for lower panels) and **(F)** quantification of fractal dimension (n = 6). All experiments were done under hyperglycemic and hypoxic conditions. Cells treated with CM-Con were used as controls. Data were presented as mean ± SD. **P < 0.01; CM-Con: conditioned medium obtained from DMSO-treated C2C12 cells, CM-Dapa: conditioned medium obtained from dapagliflozin-treated C2C12 cells.

One important feature of skeletal muscle cells is their paracrine ability to interact with other types of cell and modulate the angiogenesis process (Giudice and Taylor, 2017). At the initiation stage of angiogenesis, formation of the inner vessel tube occurs through the recruitment of vascular endothelial cells. Therefore, cellular potentials to proliferate and migrate are crucial for the whole process (Wang et al., 2019). We further explored the effect of dapagliflozin on vascular endothelial cells through skeletal muscle cells secretome. Conditioned media were obtained from dapagliflozin-treated C2C12 cells or DMSO-treated cells (CM-Dapa and CM-Con, respectively) and used for culturing HUVECs. We found that, compared with those cultured with CM-Con, the ratio of EdU-positive cells significantly increased in HUVECs treated with CM-Dapa (**Figures 6A, B**). Furthermore, CM-Dapa also increased HUVECs migration potential, as it enhanced the total number of HUVECs migrated to the lower compartment of the transwell chamber (**Figures 6C, D**), most plausibly due to the increase of F-actin polymerization in HUVECs (**Figures 6E, F**).

**Figure 7:**
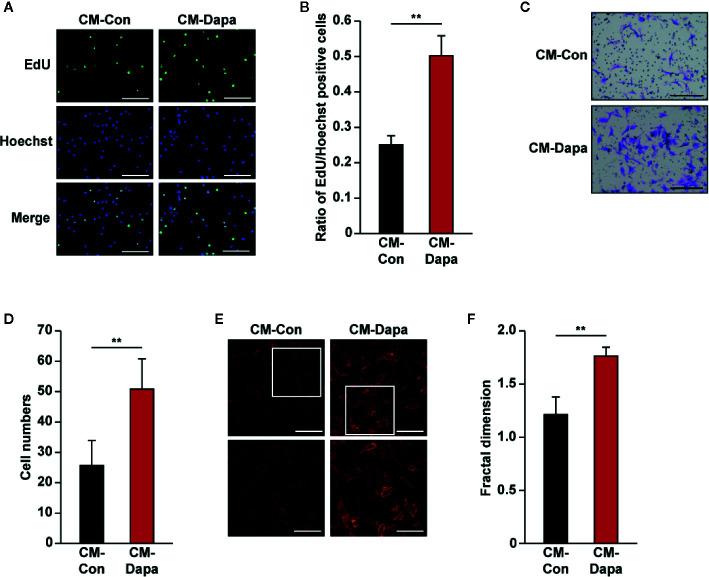
Dapagliflozin enhances smooth muscle cells’ migration and proliferation potentials under hyperglycemia through secretome of skeletal muscle cells. **(A, B)** The effect of CM-Dapa on MOVAS cells’ proliferation potential, as examined using EdU incorporation assay: **(A)** representative images (scale bars: 100 μm) and **(B)** ratio of EdU-positive cells to Hoechst-positive cells (n = 6) were shown. **(C, D)** The effect of CM-Dapa on MOVAS cells’ migration potential, as analyzed using transwell migration assay: **(C)** representative images (scale bars: 100 μm) and **(D)** quantification results (n = 6). **(E, F)** Polymerization of F-actin in MOVAS cells cultured with CM-Dapa, as analyzed using phalloidin staining: **(E)** representative images (scale bars: 100 μm for upper panels and 50 μm for lower panels) and **(F)** quantification of fractal dimension (n = 6). All experiments were done under hyperglycemic and hypoxic conditions. Cells treated with CM-Con were used as controls. Data were presented as mean ± SD. **P < 0.01; CM-Con: conditioned medium obtained from DMSO-treated C2C12 cells, CM-Dapa: conditioned medium obtained from dapagliflozin-treated C2C12 cells.

While vascular endothelial cells are crucial for tube formation, blood vessels formed by endothelial cells alone are immature and leaky. To form a mature and functional blood vessel, smooth muscle cells are recruited to cover the tube formed by vascular endothelial cells (Jain, 2003). Thus, we next investigated the effect of CM-Dapa on smooth muscle cells. Similar to its effect on HUVECs, our results showed that, compared to cells cultured with CM-Con, the ratio of EdU-positive cells significantly increased when MOVAS cells were cultured with CM-Dapa (**Figures 7A, B**). Moreover, CM-Dapa treatment also promoted the migration potential of MOVAS cells (**Figures 7C, D**), as well as F-actin polymerization (**Figures 7E, F**).

It is noteworthy that treatment with dapagliflozin did not significantly affect the angiogenic factors’ expression of HUVECs (**Figures S2A, B**). Moreover, proliferation and migration potentials of HUVECs were not altered upon treatment with 10 μM dapagliflozin (**Figures S2C–H**). Similar effects were observed in MOVAS cell lines, whereas dapagliflozin treatment failed to significantly affect the cellular expression of angiogenic factors (**Figures S3A, B**), as well as cell proliferation (**Figures S3C, D**) and migration (**Figures S3E–H**) potentials.

These findings indicated that the secretome of dapagliflozin-treated C2C12 cells improved the potentials of both vascular endothelial and smooth muscle cells to proliferate and migrate, and thus the effect of dapagliflozin on these cells was mediated by the paracrine function of skeletal muscle cells.

### Dapagliflozin Promotes Neovascularization in Diabetic HLI Mice

**Figure 8:**
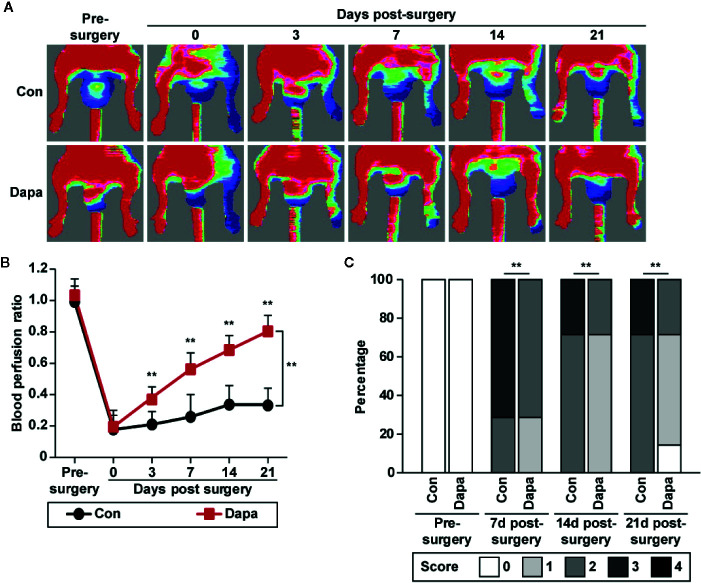
Dapagliflozin promotes blood perfusion recovery in diabetic HLI mice. **(A, B)** Blood perfusion in the ischemic hindlimbs of diabetic HLI mice administrated intramuscularly with dapagliflozin (10 mg/kg body weight) at indicated times: **(A)** representative images of Laser Doppler Imager; **(B)** blood perfusion ratio of ischemic hindlimb (left) to non-ischemic hindlimb (right). Quantification data were presented as mean ± SD (n = 7 each group). **(C)** Morphological assessment of ischemic hindlimb in diabetic HLI mice administrated intramuscularly with dapagliflozin at indicated time points (0 = no difference with control, 1 = mild change in color, 2 = moderate change in color, 3 = severe change in color, necrosis, loss of subcutaneous tissue, and 4 = lower-extremity amputations; n = 7 each group). Data were presented as mean ± SD. **P < 0.01; Con: mice administered with PBS, Dapa: mice administered with dapagliflozin.

As described above, dapagliflozin could enhance skeletal muscle cells’ paracrine function, thereby affecting the proliferation and migration potentials of vascular endothelial cells and smooth muscle cells. Therefore, we next examined whether dapagliflozin could promote neovascularization in diabetic HLI mice. Accordingly, we established diabetic HLI mice by excising the femoral artery of the left hindlimb of diabetic mice and administered dapagliflozin intramuscularly to the gastrocnemius muscle near the ischemic location, *i.e.*, near the excision location. We then measured the blood perfusion in both the left and right hindlimb using Laser Doppler Imager. The red area in the images represents areas with blood perfusion, while the blue area represents areas without blood perfusion. As shown in **Figure 8A**, compared with the control group, dapagliflozin treatment robustly increased the blood perfusion recovery in diabetic HLI mice. Moreover, blood perfusion quantification results showed a significant difference between the dapagliflozin-treated group and control group starting from day 3 after surgery (**Figure 8B**). At day 21, the blood perfusion ratio in the ischemic hindlimb had recovered to nearly 80%, while that of the control group had only recovered to less than 30% (**Figure 8B**). Ischemic damage assessment on the hindlimb further indicated the curative effect of dapagliflozin. Dapagliflozin-treated mice showed better limb morphologies, with most of the mice scoring 1, while almost all the mice in the control group scored 2 or 3 (**Figure 8C**). These results clearly demonstrated the effect of dapagliflozin in enhancing blood perfusion recovery in diabetic HLI mice.

**Figure 9:**
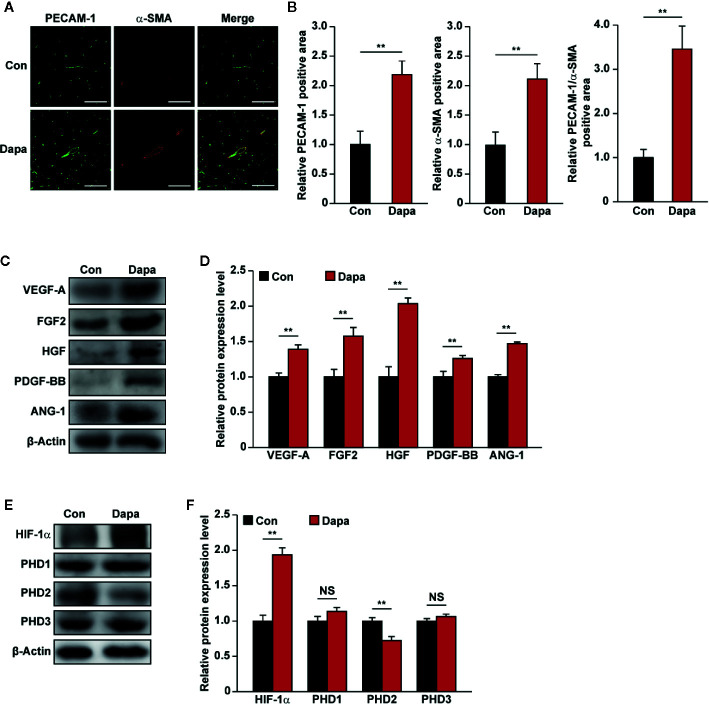
Dapagliflozin enhances neovascularization in diabetic HLI mice. **(A, B)** Immunofluorescence against PECAM-1 (green) and α-SMA (red) in ischemic hindlimbs tissue of diabetic HLI mice administrated intramuscularly with dapagliflozin at day 21 after surgery: **(A)** representative images (scale bars: 100 μm); **(B)** quantification results (n = 6) were shown. **(C, D)** The protein expression levels of angiogenic factors in the ischemic hindlimbs of diabetic HLI mice administrated intramuscularly with dapagliflozin, as examined using western blotting: **(C)** representative images and **(D)** quantification results (n = 3) were shown. **(E, F)** The protein expression levels of angiogenic factor regulators in the ischemic hindlimbs of diabetic HLI mice administrated intramuscularly with dapagliflozin, as examined using western blotting: **(E)** representative images and **(F)** quantification results (n = 3) were shown. β-Actin was used as western blotting loading control. Data were presented as mean ± SD. NS, not significant, **P < 0.01; Con: mice administered with PBS, Dapa: mice administered with dapagliflozin.

Next, we examined the mechanism underlying the blood perfusion recovery induced by intramuscular injection of dapagliflozin in diabetic HLI mice. We performed immunofluorescence staining against platelet endothelial cell adhesion molecule-1 (PECAM-1), which is a marker of vascular endothelial cells, and alpha-smooth muscle actin (α-SMA), which is a marker of smooth muscle cells. As shown in **Figure 9A**, dapagliflozin treatment conspicuously increased the number of both PECAM-1- and α-SMA-positive cells (shown in green and red, respectively). It also increased the number of PECAM-1 tube-like structures covered by α-SMA-positive cells (merged in yellow), indicating the increase of mature blood vessels (**Figure 9B**). Moreover, dapagliflozin could enhance both the PECAM-1 single positive structures, which are dot-like structures (**Figures 9A, B** left panels), as well as PECAM-1/α-SMA double-positive structures, which are larger tube-like structures (**Figures 9A, B** right panels). Given that capillaries consist of endothelial cells surrounded by a basement membrane and a sparse layer of pericytes embedded within the endothelial cells’ basement membrane, while arterioles consist of tube-like structures completely invested with smooth muscle cells (Jain, 2003; Corliss et al., 2019), these results suggest that dapagliflozin could enhance both the capillary and arteriole densities in the gastrocnemius muscle of diabetic HLI mice. Next, we demonstrated that dapagliflozin treatment robustly increased the expression levels of VEGF-A, FGF2, HGF, PDGF-BB, and ANG-1 in the gastrocnemius muscle of the ischemic hindlimb of diabetic HLI mice (**Figures 9C, D**). In line with the *in vitro* results, protein expression of PHD2, but not PHD1 and PHD3, was suppressed by dapagliflozin treatment, while HIF-1α protein accumulation increased (**Figures 9E, F**). These results suggested that intramuscularly-administered dapagliflozin could enhance the formation of mature blood vessels in diabetic HLI mice. It is noteworthy that there was no significant difference between the blood glucose level of dapagliflozin-treated group and control group (**Table S2**), indicating that the effect of dapagliflozin in promoting angiogenesis in diabetic HLI mice was due to its direct function on skeletal muscle cells, not by ameliorating the systemic hyperglycemia. Together, our results clearly indicated that intramuscular dapagliflozin administration improved the formation of mature blood vessels, and effectively promoted blood perfusion recovery in diabetic HLI model mice.

## Discussion

Diabetes is associated with vascular complications and characterized by insufficient endogenous neovascularization potential. While direct revascularization is not effective in diabetic patients, therapeutic approaches delivering angiogenic factors have arisen as one promising treatment (Jude et al., 2010). However, therapeutic angiogenesis using a single angiogenic factor could not produce an effective neovascularization due to the complexity of the angiogenesis process (Sun et al., 2011). Previous studies have reported that skeletal muscle has the ability to secrete various angiogenic factors, leading to capillary growth that supports an effective angiogenesis (Tateno et al., 2006; Wu et al., 2015; Zhang et al., 2017). Hence, inducing multiple angiogenic factors’ expression in skeletal muscle might yield a successful outcome in diabetic HLI.

Dapagliflozin is the first SGLT2 inhibitor used for anti-hyperglycemia therapy licensed by the FDA in 2014 (Meng et al., 2008; Saeed and Narendran, 2014). Besides lowering blood glucose, dapagliflozin has protective roles in diabetic renal function by suppressing apoptosis, inflammation, and endoplasmic reticulum (ER) stress (Jaikumkao et al., 2018). Intriguingly, a previous study revealed the ability of dapagliflozin to induce HIF-1α expression in renal cells (Chang et al., 2016). In this study, we demonstrated that dapagliflozin increases skeletal muscle cells’ proliferation. Moreover, dapagliflozin conspicuously increases the expression of MyoD1 and MyoG, indicating its role in stimulating skeletal muscle differentiation, whereas cells with a high expression of these factors have been shown to generate a higher differentiation potential under hypoxia (Yun et al., 2005). Indeed, previous studies showed that dapagliflozin could suppress the level of myostatin, a negative regulator of muscle growth, leading to the maintenance of skeletal muscle mass (Yamakage et al., 2020). Furthermore, we found that dapagliflozin increases the expression levels of angiogenic factors in skeletal muscle cells, such as VEGF-A, HGF, FGF2, PDGF-BB, and ANG-1. VEGF-A is the primary angiogenesis inducer, which is required to initiate neovessel formation by promoting endothelial cells’ proliferation and migration (Semenza, 2003; Liu et al., 2019), while HGF is a potent mitogen of endothelial cells which works synergistically with VEGF-A to promote endothelial cells’ function (Gerritsen, 2005). Furthermore, while VEGF-A is crucial for tube-formation, blood vessels induced merely by VEGF-A are immature and leaky, as they are not covered by the smooth muscle cells (Jain, 2003). Indeed, other angiogenic factors, including FGF2, PDGF-BB, and ANG-1, are necessary to mediate vessel maturation. FGF2 regulates multiple steps of angiogenesis, such as the extracellular matrix breakdown and induction of vascular endothelial cells’ potential to proliferate and migrate (Ferraro et al., 2010). Importantly, FGF2 also activates downstream angiogenic pathways and works in concert with PDGF-BB to induce vessel maturation (Cao et al., 2003). PDGF-BB plays a major role in vessel maturation and also promotes FGF2 release to induce smooth muscle cells’ proliferation (Millette et al., 2005; Wang et al., 2019). ANG-1 is also required for neovessel maturation as it recruits smooth muscle cells (Chen and Stinnett, 2008; McClung et al., 2015; Wang et al., 2019). It could also mediate the paracrine function between muscle and endothelial cells (McClung et al., 2015). Collectively, diabetic HLI could be ameliorated through the interplay effect between angiogenic factors secreted from skeletal muscle. As an approved anti-hyperglycemia drug, the availability and safety of dapagliflozin has been extensively shown. Hence, dapagliflozin repurposing will display several merits for further research, such as the discovery of new clinical applications and the elucidation of drug side effects in a time- and cost-effective manner (Ekins and Williams, 2011). Thus, present findings showing a novel application of dapagliflozin in promoting neovascularization in diabetic HLI gives a new perspective for dapagliflozin application, and might lead to a potential clinical use of dapagliflozin for diabetic HLI.

Despite our novel finding regarding the dapagliflozin effect on diabetic HLI, it is noteworthy that SGLT2 inhibitors demonstrated different effects on diabetic-associated vascular complications, particularly their therapeutic effects on peripheral artery diseases (Dicembrini et al., 2019). Recent studies have shown the protective effect of SGLT2 inhibitors (canagliflozin, empagliflozin, and dapagliflozin) against cardiovascular disease and death in patients with type 2 diabetes mellitus (Wiviott et al., 2019a; Arnott et al., 2020); however, oral admission of canagliflozin could increase the risk of limb amputation (Matthews et al., 2019), while both dapagliflozin and empagliflozin exhibit no evidence of risk (Dicembrini et al., 2019). Similarly, *in vivo* studies using diabetic HLI mice treated with orally administered canagliflozin results in a lower blood perfusion recovery compared to the control, while treatment with dapagliflozin results in a blood perfusion recovery comparable to that of the control (Lin et al., 2020).

Intriguingly, while dapagliflozin is known as an oral anti-hyperglycemic drug, we found that blood glucose levels of diabetic HLI mice do not change significantly after intramuscular injection of dapagliflozin. On the other hand, Lin *et al.* showed that, compared to that of the control group, oral administration of dapagliflozin does not improve blood perfusion recovery in diabetic HLI mice (Lin et al., 2020). Although the detailed mechanisms involved need to be investigated further, these distinct effects are most plausibly due to the difference in administration method. Orally-administrated dapagliflozin might be metabolized and distributed systematically, while intramuscular injection provided a more localized and direct effect of dapagliflozin. Furthermore, given that direct dapagliflozin treatment enhanced only the proliferation and migration potentials of skeletal muscle cells but not vascular endothelial and smooth muscle cells, these direct effect of dapagliflozin might be not a general one; instead, it might be an effect on specific cell types. Nevertheless, our findings provide a possibility for utilizing intramuscularly-administered dapagliflozin for achieving efficient therapeutic angiogenesis in diabetic HLI. Hence, a better understanding on how different SGLT2 inhibitors lead to different adverse effects is urgently needed. Taken together with our finding, the discrepancies among different studies indicate the need for further evaluation on the class effect of SGLT2 inhibitors, as well as the administration methods in affecting angiogenesis of diabetic HLI disease.

The PHD family, which consists of PHD1, PHD2, and PHD3, plays a major role in regulating neovascularization, particularly by inducing HIF-1α degradation. Inhibitors targeting the PHD family have been regarded as a potential approach to stimulate the angiogenesis process. However, simultaneous deletion of multiple PHD genes has been shown to induce adverse effects, such as hepatotoxicity (Taniguchi et al., 2013). The use of pan-inhibitors for PHDs could also trigger toxic effects associated with sustained HIF-1α upregulation, thereby encouraging the utilization of specific PHD inhibition (Loinard et al., 2009). In this study, we found that dapagliflozin exerts its functions in inducing HIF-1α protein accumulation and suppressing the expression of PHD2, suggesting its potent activity on PHD2/HIF-1α axis.

In response to hypoxia, HIF-1α activates numerous angiogenic factors, including VEGF-A and PDGF-BB, making it a crucial factor for angiogenesis induction (Kelly et al., 2003). However, previous studies showed that some factors might regulate HIF-1α independently (Wu et al., 2008; Chan et al., 2009; Wang et al., 2013; Wu et al., 2015). Indeed, PHD2 inhibition could induce several angiogenic factors in a HIF-1α independent manner, such as HGF, ANG1, and FGF2 (Onimaru et al., 2002; Chan et al., 2009; Wu et al., 2015). Together with the fact that dapagliflozin could still slightly affect skeletal muscle cells’ proliferation and migration potentials, as well as the expression of angiogenic factors in *HIF-1α*-silenced skeletal muscle cells, it is most likely that dapagliflozin could improve skeletal muscle cells’ angiogenic potentials in both HIF-1α-dependent and -independent manners.

Vascular endothelial cells play a key role in regulating vascular permeability, vascular growth, and angiogenesis. They also maintain the migration and proliferation potentials of smooth muscle cells (Sena et al., 2013), which stabilize and functionalize the blood vessel (Jain, 2003). In this study, we revealed that direct treatment of dapagliflozin does not affect the expression of angiogenic factors in vascular endothelial and smooth muscle cells, or their proliferation and migration potentials. Meanwhile, the angiogenic factors secreted by dapagliflozin-treated skeletal muscle cells could stimulate both vascular endothelial and smooth muscle cells’ proliferation and migration potentials. These results are consistent with those of previous studies, showing that 10 μM dapagliflozin did not affect the proliferation and migration of vascular endothelial cells (Mancini et al., 2018; Behnammanesh et al., 2019), as well as those of smooth muscle cells (Behnammanesh et al., 2020). Together, these results suggest that intramuscularly-administered dapagliflozin exerts its function in inducing neovascularization and blood perfusion recovery by promoting the paracrine function of skeletal muscle cells, which in turn enhances the proliferation and migration potentials of vascular endothelial and smooth muscle cells.

Together, while further pre-clinical and clinical studies are required to explore the possibility of utilizing dapagliflozin as a diabetic HLI drug, our study has demonstrated for the first time that intramuscularly-administered dapagliflozin could induce effective neovascularization in diabetic HLI mice, and thus opens up the possibility of using intramuscularly-administered dapagliflozin to induce neovascularization for treating diabetic HLI.

## Conclusion

By discovering new clinical applications of an oral anti-hyperglycemic drug, dapagliflozin, further research might be conducted in a time- and cost-effective manner. In this study, we revealed an unknown role of dapagliflozin in promoting neovascularization in diabetic HLI through PHD2/HIF-1α axis in skeletal muscle cells, which suggests the potential application of dapagliflozin as a therapeutic angiogenesis strategy for diabetic HLI.

## Data Availability Statement

The raw data supporting the conclusions of this article will be made available by the authors, without undue reservation, to any qualified researcher.

## Ethics Statement

The animal study was reviewed and approved by Laboratory Animal Welfare and Ethics Committee of the Third Military Medical University.

## Author Contributions

VK and SW arranged the research, designed the experiments, examined and construed the data, wrote the manuscript, provided financial support, and guided all the experiments. DAN and OM performed most of the cellular and animal experiments, examined and construed the data, and wrote the manuscript. CL executed part of the animal experiment.

## Funding

This work was supported by grants from the Fundamental Research Funds for the Central Universities (2019CDQYSW010); and the Natural Science Foundation of Chongqing (cstc2018jcyjAX0374 and cstc2018jcyjAX0411).

## Conflict of Interest

A patent related to the results of this study has been filed with Chinese patent application No. 202010701077.7.

The authors declare that the research was conducted in the absence of any commercial or financial relationships that could be construed as a potential conflict of interest.
