# Supplementary material for: Dapagliflozin Promotes Neovascularization by Improving Paracrine Function of Skeletal Muscle Cells in Diabetic Hindlimb Ischemia Mice Through PHD2/HIF-1α Axis
Source: Front Pharmacol. 2020 Aug 10;11:1104. doi: 10.3389/fphar.2020.01104 (PMC7424065; doi:10.3389/fphar.2020.01104)
Supplement: Supplementary file 1 [file DataSheet_1.pdf]

Supplementary Material

Dapagliflozin promotes neovascularization by improving paracrine function of skeletal muscle cells in diabetic hindlimb ischemia mice through PHD2/HIF-1α axis

Dyah Ari Nugrahaningrum, Olivia Marcelina, Caiping Liu, Shourong Wu*, and Vivi Kasim*

*Email: vivikasim@cqu.edu.cn (V.K.)
shourongwu@cqu.edu.cn (S.W.)

Figure S1

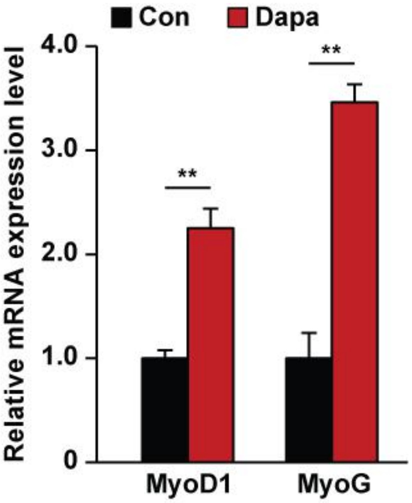

FIGURE S1 | The mRNA expression level of skeletal muscle differentiation factors in C2C12 cells treated with dapagliflozin. MyoD1 and MyoG mRNA expression levels of C2C12 cells treated with dapagliflozin under hyperglycemia, as determined using reverse-transcription PCR. β-Actin was used for normalization. Data were shown as relative to that of control and expressed as mean ± SD. ***P* < 0.01; Con: DMSO-treated C2C12 cells, Dapa: dapagliflozin-treated C2C12 cells.

Figure S2

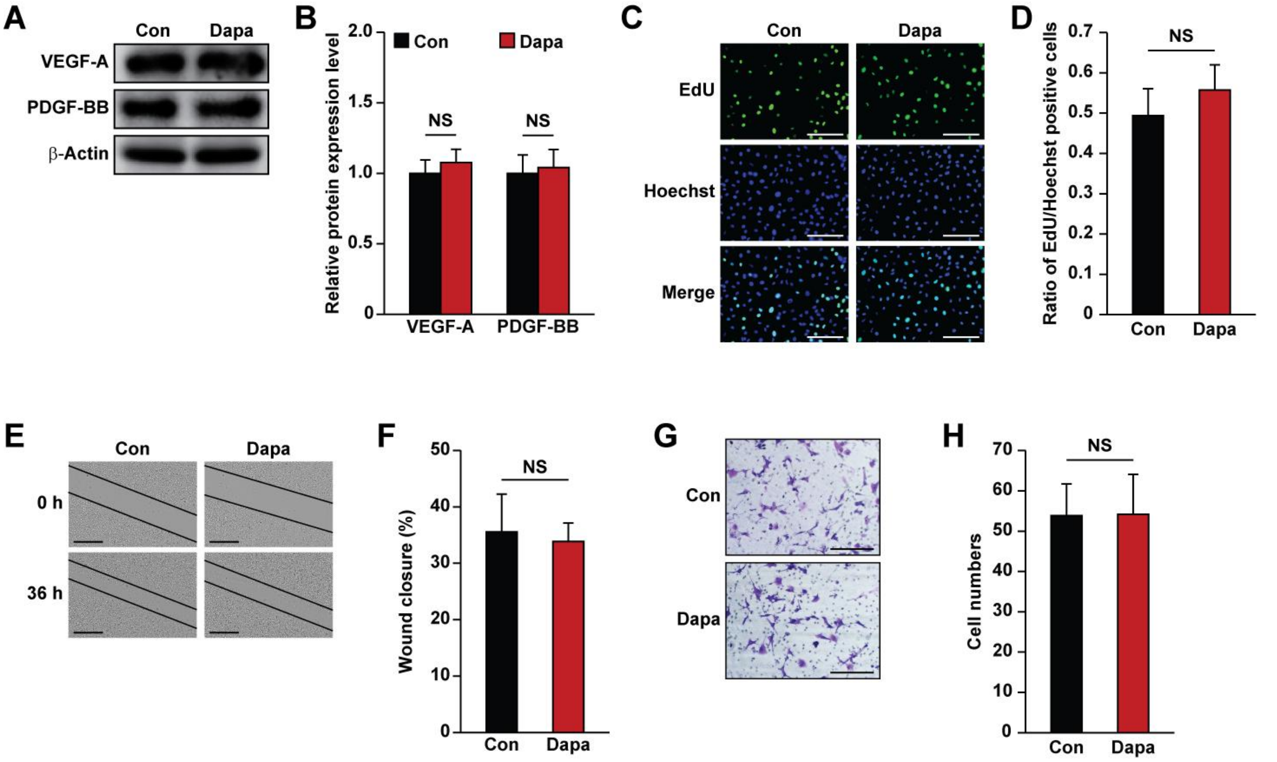

FIGURE S2 | The effect of dapagliflozin on angiogenesis potential of vascular endothelial cells under hyperglycemia. **(A, B)** The expression levels of angiogenic factors protein in HUVECs treated with dapagliflozin (final concentration: 10 μM). Protein levels were examined using western blotting: **(A)** representative images and **(B)** quantification results (n = 3) were shown. **(C, D)** The effect of dapagliflozin treatment (final concentration: 10 μM) on HUVECs proliferation potential as evaluated by EdU incorporation assay: **(C)** representative images (scale bars: 100 μm) and **(D)** ratio of EdU-positive cells to Hoechst-positive cells (n = 6) were shown. **(E, F)** Migration potential of HUVECs after dapagliflozin treatment (final concentration: 10 μM), as analyzed by scratch assay: **(E)** representative images (scale bars: 200 μm) and **(F)** quantification of wound closure rate (n = 6) were shown. **(G, H)** Migration potential of HUVECs after dapagliflozin treatment (final concentration: 10 μM), as investigated using transwell migration assay: **(G)** representative images (scale bars: 100 μm) and **(H)** quantification results (n = 6) were shown. All experiments were done under hyperglycemic and hypoxic condition. Cells treated with DMSO were used as controls. β-actin was used as a loading control for western blotting. Quantification data were presented as mean ± SD. NS: not significant; Con: DMSO-treated HUVECs, Dapa: dapagliflozin-treated HUVECs.

Figure S3

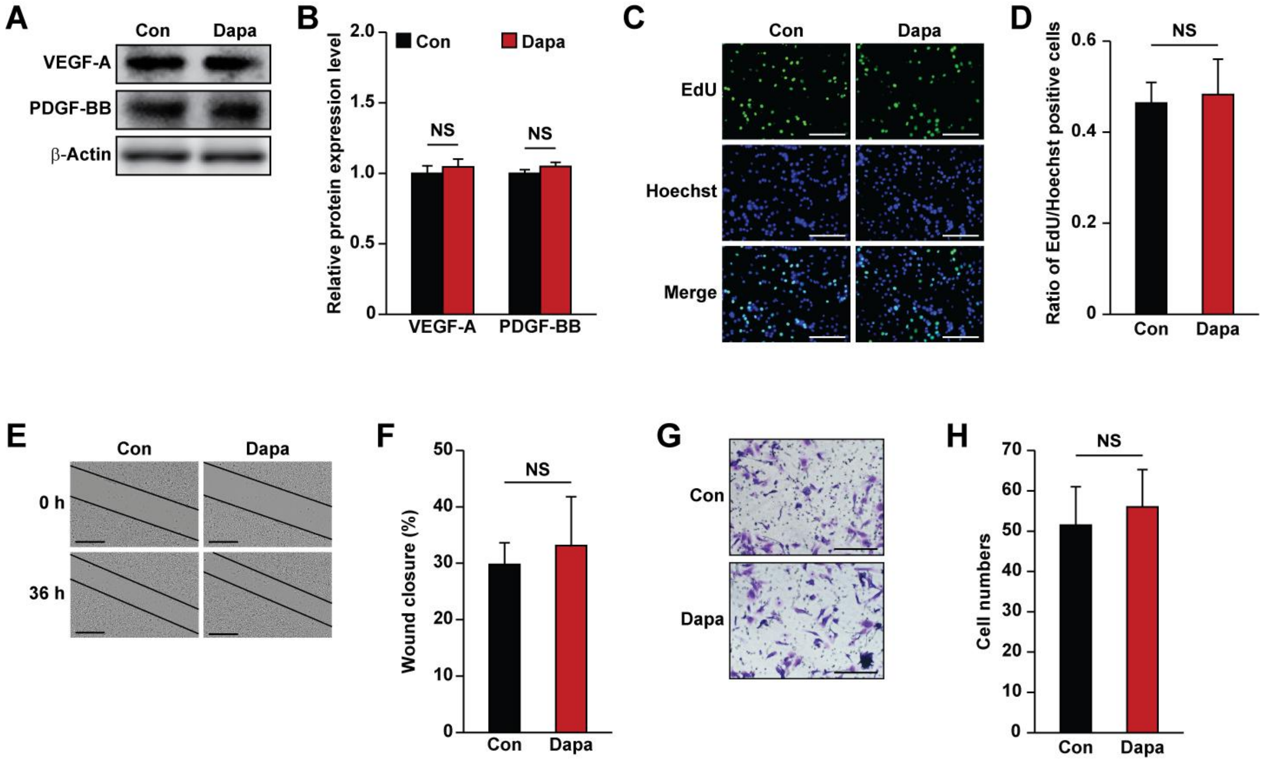

FIGURE S3 | The effect of dapagliflozin on angiogenesis potential of smooth muscle cells under hyperglycemia. **(A, B)** The expression levels of angiogenic factors protein in MOVAS cells treated with dapagliflozin (final concentration: 10 μM). Protein levels were examined using western blotting: **(A)** representative images and **(B)** quantification results (n = 3) were shown. **(C, D)** The effect of dapagliflozin treatment (final concentration: 10 μM) on MOVAS cells proliferation potential as evaluated by EdU incorporation assay: **(C)** representative images (scale bars: 100 μm) and **(D)** ratio of EdU-positive cells to Hoechst-positive cells (n = 6) were shown. **(E, F)** Migration potential of MOVAS cells after dapagliflozin treatment (final concentration: 10 μM), as analyzed by scratch assay: **(E)** representative images (scale bars: 200 μm) and **(F)** quantification of wound closure rate (n = 6) were shown. **(G, H)** Migration potential of MOVAS cells after dapagliflozin treatment (final concentration: 10 μM), as investigated using transwell migration assay: **(G)** representative images (scale bars: 100 μm) and **(H)** quantification results (n = 6) were shown. All experiments were done under hyperglycemic and hypoxic condition. Cells treated with DMSO were used as controls. β-actin was used as a loading control for western blotting. Quantification data were presented as mean ± SD. NS: not significant; Con: DMSO-treated MOVAS cells, Dapa: dapagliflozin-treated MOVAS cells.

TABLE S1 | Antibodies and chemicals used for western blotting, immunofluorescence, and phalloidin staining.

Antibody	Product No.	Company	Experiment	Dilution
anti-HIF-1α	NB100-449	Novus Biological	Western blotting	1/2000
anti-PHD1	NB100-310	Novus Biological	Western blotting	1/1000
anti-PHD2	NB100-138	Novus Biological	Western blotting	1/1000
anti-PHD3	Ab184714	Abcam	Western blotting	1/2000
anti-VEGF-A	sc-152	Santa Cruz Biotechnology	Western blotting	1/300
anti-PDGF-BB	sc-7878	Santa Cruz Biotechnology	Western blotting	1/300
anti-HGF	sc-7949	Santa Cruz Biotechnology	Western blotting	1/200
anti-FGF2	sc-79	Santa Cruz Biotechnology	Western blotting	1/500
anti-ANG-1	AB10516	Millipore	Western blotting	1/1000
anti-β-Actin	60008-l-Ig	Protein Tech	Western blotting	1/20000
Goat Anti-Mouse IgG	ZB2305	ZSGB-BIO	Western blotting	1/10000
Goat Anti-Rabbit IgG	ZB2301	ZSGB-BIO	Western blotting	1/10000
anti-PECAM-1	550274	BD Pharmingen	Immunofluorescence	1/100
Monoclonal anti-murine α-SMA Cy3 conjugate	C6198	Sigma-Aldrich	Immunofluorescence	1/100
Alexa Fluor 488 Goat Anti-Rat IgG	A11006	Invitrogen	Immunofluorescence	1/500
Alexa Fluor 555 Phalloidin	A34055	Invitrogen	Phalloidin staining	1/250
Hoechst 33342	C0071S-6	Beyotime	EdU incorporation assay	1/1000

TABLE S2 | Blood glucose concentration in diabetic hindlimb ischemia model mice during the experiment.

Control (mmol/l)				Dapagliflozin (mmol/l)			
**No**	**3 weeks HFD**	**1 day pre-surgery**	**3 weeks post-surgery**	**No**	**3 weeks HFD**	**1 day pre-surgery**	**3 weeks post-surgery**
1	8.5	20.8	20	1	6.4	24.86	24.4
2	6	20	19.8	2	6.3	25.5	23.5
3	6.8	21.8	17.6	3	6	21.5	21.1
4	7.1	17.8	17.4	4	4.8	21.4	20
5	6	18.4	17.7	5	6.8	22.1	19.1
6	5.7	19.4	18.3	6	5	23.1	20.4
7	5.3	23	22	7	5.7	19.9	17.6
**Mean**	**6.49**	**20.17**	**18.97**	**Mean**	**5.86**	**22.62**	**20.87**
**Stdev**	**1.08**	**1.85**	**1.70**	**Stdev**	**0.68**	**1.85**	**2.21**
***P* value**		**1.E-09$^{\#}$**	**0.230***	***P* value**		**8.71E-11$^{\#}$**	**0.163***

HFD: high fat diet

$^{\#}$*P* value was calculated versus 3 weeks HFD using one-way ANOVA.

**P* value was calculated versus 1 day pre-surgery using one-way ANOVA.
